# The role of DAAO in cognitive impairment of offspring mice induced by arsenic exposure during early developmental stage

**DOI:** 10.1371/journal.pone.0333414

**Published:** 2025-09-29

**Authors:** Zehua Niu, Xiao Xie, Xiaoxia Jin, Haiyang Yu, Ge Jin, Yan Wang

**Affiliations:** 1 Department of Occupational and Environmental Health, School of Public Health, Shenyang Medical College, Shenyang, Liaoning, People’s Republic of China; 2 Key Laboratory of Environment and Population Health of the Educational Department of Liaoning Province, Shenyang, People’s Republic of China; 3 Department of Toxicology, School of Public Health, Shenyang Medical College, Shenyang, Liaoning, People’s Republic of China; 4 Department of pharmacology, School of Traditional Chinese Medicine, Shenyang Medical College, Shenyang, Liaoning, People’s Republic of China; Northwest Institute of Plateau Biology Chinese Academy of Sciences, CHINA

## Abstract

Arsenic exposure model of offspring mice was established and intervened with 6-chlorobenzo[d]isoxazol-3-ol (CBIO), a D-amino acid oxidase (DAAO) inhibitor, to explore the role of DAAO in cognitive impairment of offspring mice induced by arsenic during early developmental stage. Female mice and their pups treated with 0 or 60 mg/L sodium arsenite (NaAsO_2_) via drinkable water from the first day of gestation till the end of lactation. On the 28th day after birth, the offspring mice in the drinking distilled water group were randomly divided into control and 1 mg/mL CBIO group. The offspring mice in the arsenic group were divided into 60 mg/L NaAsO_2_ group and 60 mg/L NaAsO_2_ + 1 mg/mL CBIO group, CBIO was administered to the lateral ventricle for one week. Additionally, D-serine and L-serine concentrations were detected by UHPLC-MS/MS, Real-time RT-PCR and Western blot were applied to measure DAAO, serine racemase (SR), N-methyl-D-aspartate receptor (NMDAR), synaptophysin (SYP) and postsynaptic density (PSD95) levels in the hippocampus. Results disclosed that arsenic could reduce the levels of D-serine, L-serine, SR and NMDAR, while upregulate DAAO levels, however, inhibiting DAAO levels could increase D-serine and NR1 levels. These findings indicated that DAAO might be involved in cognitive impairment of offspring mice induced by arsenic during early developmental stage by affecting D-serine metabolism.

## Introduction

Arsenic is found in many parts of nature, and humans mostly consume it in the form of drinking water and food. In many regions around the world, the concentration of inorganic arsenic in drinking water has exceeded the standards set by the World Health Organization, seriously endangering millions of people`s health worldwide. Prolonged intake of arsenic can lead to multi-system diseases, such as the nervous system, cardiovascular system, and typical skin symptoms [1–3]. With the deepening of research on endemic arsenic poisoning, attention has gradually turned to the impact of environmental arsenic exposure on the central nervous system (CNS) and its underlying mechanisms. Epidemiological surveys showed long-term consumption of arsenic-contaminated water could significantly affect children’s intellectual development, leading to behavioral abnormalities and intellectual impairment [4]. Signes-Pastor et al. [5] investigated the association between prenatal maternal urinary arsenic concentrations and childhood cognitive abilities, and found that arsenic exposure during pregnancy may elevate the risk for impaired cognitive performance in children. Chen et al. [6] identified inverse associations between prenatal arsenic exposure, especially in early pregnancy, and neurodevelopment of children at two years old, even at low exposure levels. The results of animal experiments showed that long-term arsenic exposure could reduce the cognitive ability [7–9]. However, the mechanism of arsenic induced cognitive impairment is not completely clear. The graphical abstract was shown in S1 Fig.

The dynamic interplay between neuronal and glial networks critically regulates higher-order cognitive functions in the mammalian brain. As the principal cellular substrate for information processing, synaptic plasticity underlies memory formation and learning processes. Electron microscopy studies reveal that astrocytic processes envelop synaptic clefts in the hippocampus, forming tripartite synapses through intimate structural interactions with presynaptic and postsynaptic elements [10,11]. In this structure, the neurotransmitters released from the presynaptic membrane act on the corresponding receptors on the postsynaptic membrane, while binding to the corresponding receptors on the astrocytes (AST) membrane, thereby affecting the changes in calcium ion signals within the AST. Through a series of signal transduction, AST releases gliotransmitters such as glutamate (Glu) and D-serine, which enter the synaptic cleft and bind to the corresponding receptors on the postsynaptic membrane, affecting the production of related reactions by the postsynaptic membrane receptors, altering synaptic transmission efficiency, and regulating synaptic plasticity [12,13]. Our preliminary data demonstrated that arsenic exposure significantly impairs AST functionality, leading to dysregulated D-serine release, suggesting a potential mechanistic link between arsenic neurotoxicity and disrupted D-serine homeostasis [14]. Furthermore, astrocyte conditioned medium (ACM) was added to the culture system of neuron to investigate the impact of arsenic-exposed AST on signal transduction within neurons, and our previous research data disclosed that the effect of arsenic on AST might indirectly lead to abnormal level of N-methyl-D-aspartate receptor (NMDAR) subunits NR1, NR2A, and NR2B proteins in neurons, suggesting that arsenic might interfere with NMDAR protein levels in neurons and the secretion of D-serine by AST might be involved in it [15].

NMDAR is a crucial ionotropic Glu receptor that is involved in memory and learning processes [16,17]. After D-serine combines with the glycine binding site of NMDAR, NMDAR coupled ion channel can be opened by Glu, thus exerting its biological effects [18,19]. Research indicated that degradation of D-serine could block the production of long-term potentiation (LTP), while administration of D-serine could obviously improve the induction and maintenance of LTP [20]. Serine racemase (SR) is the main enzyme catalyzing the synthesis of D-serine, converting L-serine into D-serine through racemization. L-serine is considered to play an indispensable role in brain, and a lack of L-serine can result in serious problems and manifests as varying degrees of neurological abnormalities [21]. The main pathway for D-serine metabolism is oxidative degradation by D-amino acid oxidase (DAAO), producing hydroxypyruvic acid (HPA) and ammonia, which are eventually synthesized into glucose through gluconeogenesis [22]. DAAO is mainly located in AST [23,24]. Studies have shown that LTP mediated by NMDAR in the hippocampus of DAAO gene-deficient mice is significantly enhanced [25]. 6-Chlorobenzo[d]isoxazol-3-ol (CBIO) is an effective inhibitor of DAAO [26]. In a murine model of closed head injury (24h post-trauma), CBIO administration notably enhanced cognitive performance, and attenuated neuroinflammation; while concomitantly upregulating hippocampal NR1 subunit expression [27].

Our previous findings indicated that the hippocampal NMDAR and D-serine levels noticeably decreased, while DAAO levels obviously increased in offspring mice exposed to arsenic, suggesting that changes of D-serine levels regulated by DAAO might be important in arsenic induced cognitive impairment [28]. Nevertheless, the specific mechanisms of DAAO in cognitive impairment induced by arsenic are not yet clear. The arsenic exposure model of offspring mice was established and intervened with CBIO to explore the role of DAAO in cognitive impairment of offspring mice induced by arsenic exposure during early developmental stage, and to explore the possible mechanisms underlying the effects of early-life arsenic exposure on the neurodevelopment of mice.

## Materials and methods

### Animal model establishment

Animal selection, breeding conditions and establishment of arsenic exposure models were described in detail in previous reported article [28]. On the first day of gestation, twenty-four pregnant Kunming mice were randomly separated into distilled water drinking group (12 mice) and 60 mg/L arsenic exposure group (12 mice). Female mice and their pups treated with 0 or 60 mg/L sodium arsenite (NaAsO_2_) via drinkable water from the first day of gestation till the end of lactation. Brain stereotactic locator was used to place a cannulae in the brain of offspring mice on the 21th day after birth, and CBIO was injected to the lateral ventricle of offspring mice on the 28th day after birth for one week according to the following groups. The offspring mice from the distilled water group were divided into control and 1 mg/mL CBIO group, the offspring mice from the arsenic exposure group were randomly divided into 60 mg/L NaAsO_2_ group and 60 mg/L NaAsO_2_ + 1 mg/mL CBIO group. On the 21th day after birth, mice were placed into the induction box of a small animal anesthesia machine (RWD510, RWD, China) with an isoflurane concentration of 5% and a flow rate of 1.5 L/min during induction. After two minutes of induction, the mice were fixed using a stereotaxic brain locator (68025, RWD, China). During mask anesthesia, the concentration of isoflurane was 1.5% and the flow rate was 0.4 L/min. The trocar was positioned and fixed according to X-1.1 mm and Y-0.5 mm. After one week of surgery, medication was injected through a trocar for intervention. The control and 60 mg/L NaAsO_2_ group were given 0.5% sodium carboxymethylcellulose (CMC) by lateral ventricle injection. The induction anesthesia conditions for small animal anesthesia machine were isoflurane concentration of 5%, flow rate of 1 L/min. After induction for 2 min, face mask anesthesia was used with isoflurane concentration of 1.5%, flow rate of 0.4 L/min. The mice were fixed and injected with drugs or solvents through a trocar using a microinjector. The injection volume was 2 μL, the injection speed was 1 μL/min, and the residence time was 3 min. Six offspring mice in per group (from different litters) were selected for each experimental project, and brain tissues were removed and the hippocampal tissues were isolated. This study procedure was approved by Scientific Research Committee of Shenyang Medical College (SYYXY2021030302) and followed the Chinese National Guidelines for the protection of laboratory animals.

### Y-Maze test

After one week of intervention in the lateral ventricle, a Y-maze experiment was conducted to measure the spatial memory function of mice. The test box (self-made) was a three-arms maze with arm length of 40 cm, width of 10 cm, and height of 10 cm. Prior to experimental procedures, mice were acclimatized to the testing environment for a minimum of 30 minutes to minimize stress-induced variability. The mouse was placed at the end of one of the arms with its face facing the outside of the maze and allowed to move freely for 5 min. The total number of times the mouse entered the arm and the spontaneous alternation rate were used as observation indicators. The mice were placed into three arms in order and marked as alternating once. The calculation formula is: % spontaneous alternation percentage (SAP) = ({spontaneous alternation/(total number of arm entries −2)} × 100). The Y-maze has three tracks, namely track A, B, and C. The mice were placed at the end of the same track in batches, and the trajectory path of each mouse was observed and recorded for 5 min. The testing apparatus was meticulously cleaned with 70% ethanol between trials to eliminate olfactory cues that could influence subsequent animal behavior. Blinding was used in this experiment.

### Hematoxylin-eosin (HE) staining

The detailed processes described by Zheng et al. [29]. Following cardiac perfusion, brain tissues were collected from different groups of neonatal mice, fixed in 4% paraformaldehyde, processed through dehydration and paraffin embedding, sectioned, dewaxed, rehydrated, stained with hematoxylin and eosin, re-dehydrated, and mounted with neutral gum for observation and image acquisition under pathological section scanner (HS50, Leica Microsystems, Germany). Cell counting was performed manually under blinded conditions to eliminate observer bias.

### Ultrahigh-performance liquid chromatography tandem triple quadrupole mass spectrometry (UHPLC-MS/MS)

Hippocampal tissue samples were prepared using acetonitrile precipitation method. 100 μL homogenized tissue was added, along with 50 μL epistine, 100 μL water, and 300 μL acetonitrile. After vortexing for 1 min, the mixture was centrifuged at 13000 rpm for 5 min. The supernatants were then nitrogen blown and filtered through 150 μL of methanol water solution. The mixture was then analyzed by UHPLC-MS/MS to detect D-serine and L-serine concentrations. Agilent 1290UHPLC liquid chromatography and Agilent 6460 QQQ mass spectrometer (Agilent, America).

The chromatographic conditions: Agilent Poroshell EC-C18 column (100 × 2.1 mm, 1.9 μm); the injection volume was 10 μL; the column temperature was 35 °C; the flow rate was 0.3 mL/min; gradient elution: mobile phase A contains a 0.1% formic acid methanol solution, mobile phase B contained a 13 mmol/L ammonium acetate aqueous solution, and mobile phase A was 2% −2.5% for 0–5 min, 2.5% for 5–6 min, 2.5% −60% for 6–6.5 min; 60% −2% for 6.5–12 min; Stop time was 12 min.

Mass spectrometry conditions: multiple reaction monitoring (MRM) was used, with positive ion scanning mode gas temperature at 325 °C; gas flow was 6 L/min; sheath gas temperature was 350 °C; sheath gas flow was 12 L/min.

### Quantitative real‑time RT‑PCR

Detailed processes described in previous study was modified [28]. The extraction of total RNA from hippocampal tissues of mice was performed using Trizol reagent (Invitrogen, USA). Furthermore, the cDNA was used as templates for real-time PCR amplification using SYBR Premix Ex Taq II (Takara, Japan) and 7500 fast real-time PCR system (7500Fast, ABI, USA). To amplify a fragment of DAAO, SR, NR1, NR2A, NR2B, SYP, PSD95 and GAPDH. Primer sequences were offered in S1 Table. Amplification was performed for 40 cycles of 3 s at 95 °C and 30 s at 60 °C. Data were analyzed using the comparative Ct method. RNA abundance were expressed as 2^−ΔΔCt^ for the target mRNA relative to those of GAPDH gene, and presented as fold change comparing to control.

### Western bolt analysis (WB)

The processes described in detail in previous study [28]. The hippocampus of mice was homogenized and protein levels were detected by BCA protein assay kit (Pierce, Rockford, IL, USA). 30 μg total protein were resolved. Antibodies were DAAO (1:500), NR1 (1:500) (Boster, China), SR (1:500), NR2A (1:500), NR2B (1:500) (Proteintech, China), SYP (1:1000), PSD95 (1:1000) (Abcam, UK), β-actin (1:10000) (Santa Cruz, USA). An image analyzing software applied to evaluate the intensity of each band (Gel-Pro analyzer v4.0) and the results were adjusted to β-actin intensity. The original uncropped and unadjusted blot images were shown in S1 File.

### Statistical analysis

IBM SPSS version 22.0 was adopted for statistical analysis. Data were represented as mean ± SD. Using one-way ANOVA to analyze group mean differences and SNK method to perform multiple comparisons. A significance level of *P* < 0.05 was considered statistically significant.

## Results

### Changes in body, brain and hippocampal weights after arsenic exposure

There were no obvious differences in body (F = 0.924, *P* = 0.447), brain (F = 0.909, *P* = 0.454), and hippocampal weights among groups (F = 0.687, *P* = 0.571) (S2 Table). The data of body, brain and hippocampal weights were shown in S2 File.

### Influence of arsenic and CBIO intervention on spatial memory

**Fig 1:**
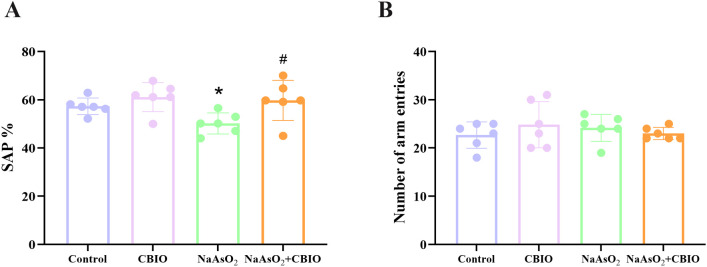
Influence of arsenic and CBIO intervention on spatial memory. **(A)** Changes of spontaneous alternation percentage in Y-maze. **(B)** Alterations of the number of arm enters in Y-maze. Data were shown as mean *± *SD, n = 6. **P* *< 0.05, ^*^ vs. the control, ^#^ vs. NaAsO_2_ group.

Significant intergroup differences were observed in the alternation reaction rate (F = 4.164, *P* = 0.019). The results of Y maze showed that the alternation reaction rate of the offspring mice exposed to arsenic was obviously lower than that in control, while CBIO intervention notably improved the alternation reaction rate of mice treated with arsenic. Otherwise, there was no difference in the total number of pups entering the arms among groups (F = 0.614, *P* = 0.614) (Fig 1).

### Effects of arsenic and CBIO intervention on hippocampal neuronal cells

**Fig 2:**
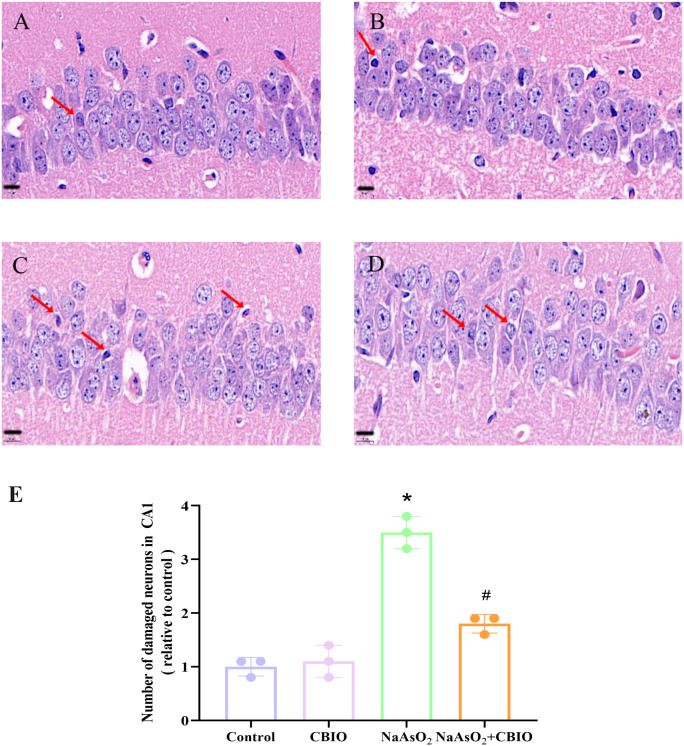
Influence of arsenic and CBIO intervention on neuronal cells in hippocampal CA1. HE staining, micrographs were captured by 100 × , scale bar = 10 μm. The arrow points to damaged neurons. **(A)** Control **(B)** CBIO group **(C)** NaAsO_2_ group **(D)** NaAsO_2_ + CBIO group **(E)** Quantitative analysis of HE results. Data were shown as mean ± SD, n = 3. **P* *< 0.05, ^*^ vs. the control, ^#^ vs. NaAsO_2_ group.

**Fig 3:**
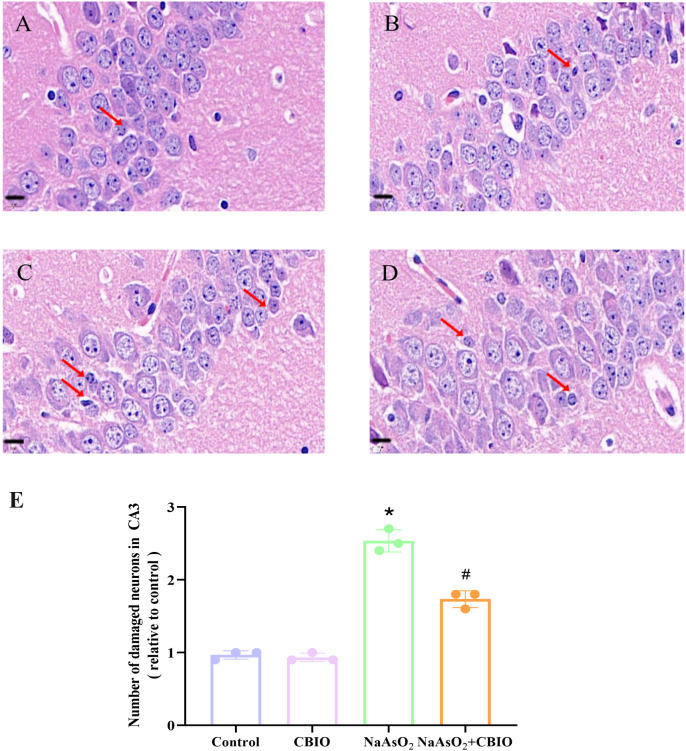
Influence of arsenic and CBIO intervention on neuronal cells in hippocampal CA3. HE staining, micrographs were captured by 100 × , scale bar = 10 μm. The arrow points to damaged neurons. **(A)** Control **(B)**CBIO group **(C)** NaAsO_2_ group **(D)** NaAsO_2_ + CBIO group **(E)** Quantitative analysis of HE results. Data were shown as mean ± SD, n = 3. **P* *< 0.05, ^*^ vs. the control, ^#^ vs. NaAsO_2_ group.

As shown in Fig 2 and Fig 3, HE staining of hippocampal CA1 and CA3 showed that cell morphology damaged, cells fragmented and the numbers of damaged neurons increased in NaAsO_2_ group, however, the numbers of damaged neurons reduced, cell morphology improved after CBIO intervention. Obvious intergroup differences were observed in the numbers of damaged neurons in hippocampal CA1 (F = 66.833, *P* = 0.0005) and CA3 (F = 158.846, *P* = 0.0002).

### Changes of SYP and PSD95 levels in the hippocampus of offspring mice

**Fig 4:**
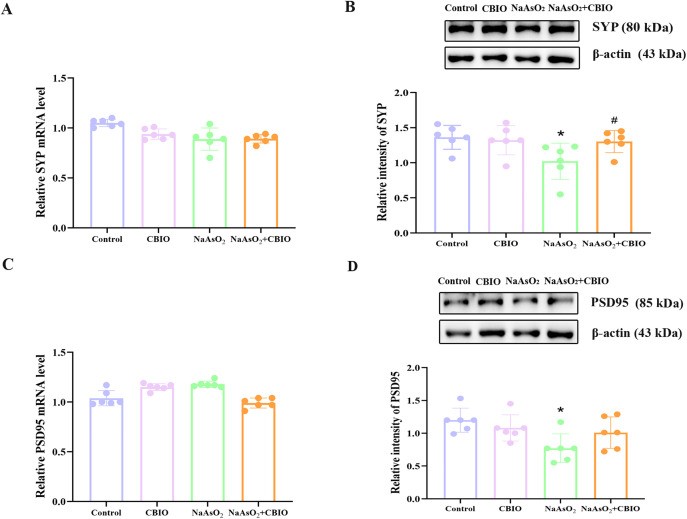
Changes of SYP and PSD95 levels in the hippocampus. **(A, C)** Real-time RT-PCR was applied to detect SYP and PSD95 mRNA levels in the hippocampus. **(B, D)** Levels of SYP and PSD95 protein were measured by WB. Images were the representative blots and statistical analysis of protein levels. Data were shown as mean ± SD, n = 6. **P* *< 0.05, ^*^ vs. the control, ^#^ vs. NaAsO_2_ group.

Significant intergroup differences were observed in the protein levels of SYP (F = 3.535, *P* = 0.033) and PSD95 (F = 4.342, *P* = 0.016). Exposure to arsenic evidently suppressed SYP and PSD95 protein levels in the hippocampus, while CBIO intervention obviously increased protein levels of SYP. However, no differences were found in the mRNA levels of SYP (F = 0.366, *P* = 0.778) and PSD95 (F = 1.417, *P* = 0.267) among groups (Fig 4).

### Effects of arsenic and CBIO intervention on D-serine and L-serine levels in the hippocampus

**Fig 5:**
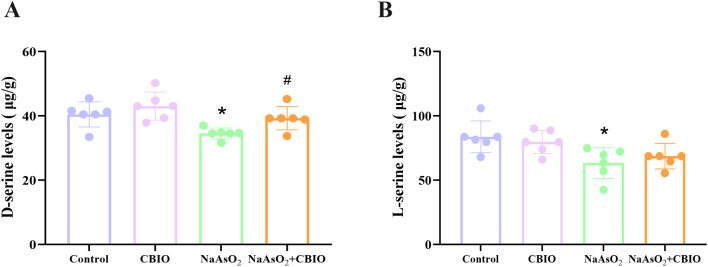
Effects of arsenic exposure and CBIO intervention on D-serine and L-serine levels in the hippocampus. **(A)** D-serine levels detected by UHPLC-MS/MS. **(B)** L-serine levels measured by UHPLC-MS/MS. Data were shown as mean ± SD, n = 6. **P* *< 0.05, ^*^ vs. the control, ^#^ vs. NaAsO_2_ group.

Significant intergroup differences were observed in both D-serine (F = 6.071, *P* = 0.004) and L-serine (F = 4.515, *P* = 0.014) concentrations. The concentrations of D-serine and L-serine in NaAsO_2_ group reduced obviously than those in control, while CBIO intervention significantly elevated D-serine concentrations, but L-serine levels had no obvious changes (Fig 5).

### Effects of arsenic and CBIO intervention on the levels of DAAO and SR in the hippocampus

**Fig 6:**
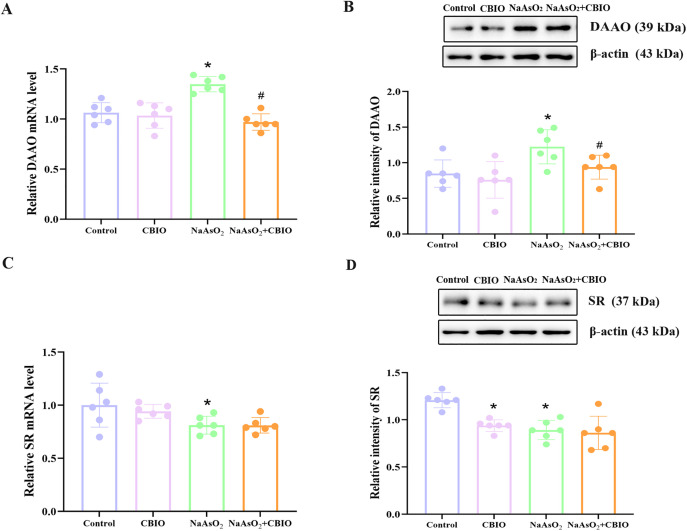
Effects of arsenic exposure and CBIO intervention on the levels of DAAO and SR. **(A, C)** Real-time RT-PCR was applied to detect DAAO and SR mRNA levels. **(B, D)** Protein levels of DAAO and SR were measured by WB. Images were the representative blots and statistical analysis of protein levels. Data were shown as mean ± SD, n = 6. **P* *< 0.05, ^*^ vs. the control, ^#^ vs. NaAsO_2_ group.

Significant intergroup differences were observed in the levels of DAAO mRNA (F = 4.471, *P* = 0.015) and protein (F = 5.170, *P* = 0.008), and SR mRNA (F = 7.7, *P* = 0.001) and protein (F = 11.684, *P* = 0.0001). Compared to control, the mRNA and protein levels of DAAO in mice treated with arsenite were obviously higher, while the levels of SR decreased obviously. Furthermore, the levels of DAAO in mice exposed to arsenite and intervened with CBIO reduced noticeably, but SR levels showed no significant differences (Fig 6).

### Effects of arsenic and CBIO intervention on the levels of NR1, NR2A and NR2B in the hippocampus

**Fig 7:**
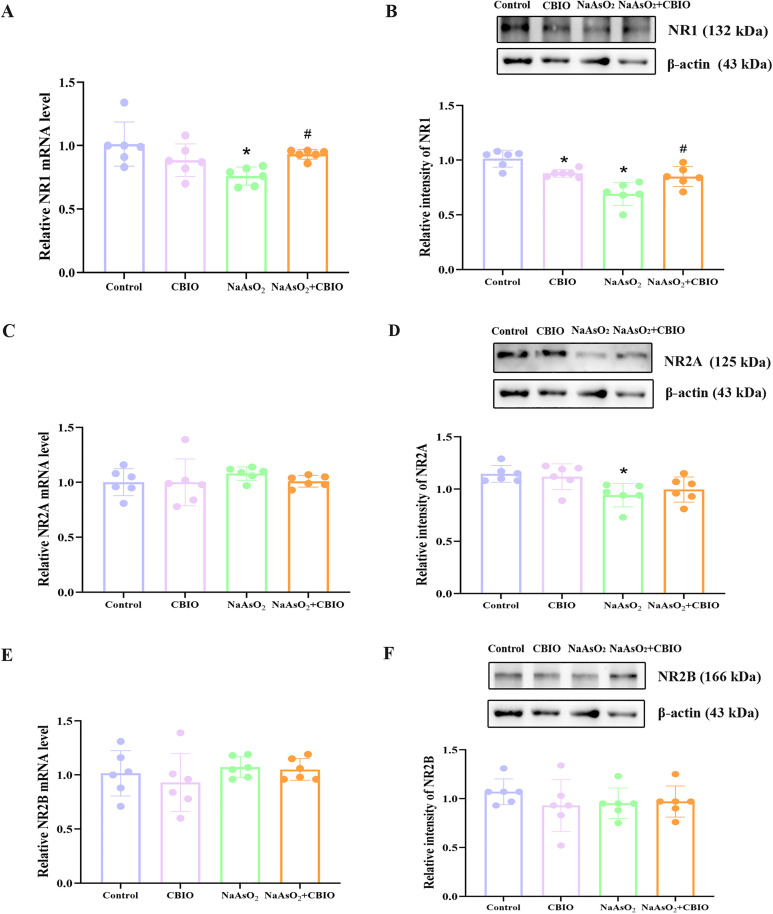
Effects of arsenic exposure and CBIO intervention on the levels of NR1, NR2A and NR2B. (A, C, **E)** Real-time RT-PCR was applied to detect NR1, NR2A and NR2B mRNA levels. (B, D, **F)** Protein levels of NR1, NR2A and NR2B were measured by WB. Images were the representative blots and statistical analysis of protein levels. Data were shown as mean ± SD, n = 6. **P* *< 0.05, ^*^ vs. the control, ^#^ vs. NaAsO_2_ group.

Obvious intergroup differences were observed in the levels of NR1 mRNA (F = 5.528, *P* = 0.006) and protein (F = 15.733, *P* = 0.0002), and NR2A protein (F = 4.690, *P* = 0.012). Exposure to arsenic obviously inhibited mRNA and protein levels of NR1, only suppressed protein levels of NR2A, while the levels of NR2A mRNA (F = 0.236, *P* = 0.870) and NR2B mRNA (F = 1.588, *P* = 0.224) and protein (F = 0.663, *P* = 0.585) had no difference among groups. Moreover, CBIO intervention obviously increased NR1 levels in mice exposed to arsenite (Fig 7).

## Discussion

Results of this study showed that there were no obvious differences in body, brain and hippocampal weights among groups, suggesting that arsenic exposure and CBIO intervention may have no effect on the growth and development of the offspring mice. The Y-maze paradigm is widely employed to assess spatial working memory in rodents. Study reported that arsenic could obviously decrease the alternation reaction rate in Y-maze [30]. Furthermore, the alternation response rate of offspring mice exposed to arsenic decreased notably, which suggested that arsenic could impair spatial memory. Moreover, CBIO intervention significantly enhanced the alternation response rate, suggesting that CBIO intervention could alleviate the spatial memory function of mice treated with arsenic. Furthermore, there was no obvious significance in the total number of entries into the branch arms among groups, indicating that the spontaneous activity of mice was not affected. Meanwhile, the observation of hippocampal neuronal cells by HE staining showed that arsenic exposure damaged the cell morphology and fragmented the neurons, whereas which could be improved by CBIO intervention. The sample size was calculated using power analysis while adhering to the 3R principles and following established protocols from previous studies, but it has certain limitations. While the current sample size enabled preliminary characterization of key parameters, future studies with expanded samples will be required to elucidate the underlying mechanisms.

Synapses are the bridges for information transmission between neurons. PSD95 and SYP are synaptic marker proteins [31]. Calcium-binding protein SYP is found in the vesicles of the presynaptic membrane and is intimately associated with memory, learning, and synaptic plasticity [32]. PSD95 is the main scaffolding protein of the postsynaptic density, playing a crucial role in maintaining synaptic structure and function, as well as in information transmission [33]. The two classic proteins are associated with learning, memory, and cognitive functions. Studies demonstrated that arsenic could considerably lower PSD95 and SYP levels [7,34]. Data of this research showed that arsenic significantly reduced SYP and PSD95 protein levels, and protein levels of SYP increased obviously after CBIO intervention, suggesting that arsenic exposure could affect the translational levels of synapse-related proteins SYP and PSD95, and SYP levels could be improved by CBIO intervention.

D-serine is a necessary endogenous co-agonist for the activation of NMDAR, which is vital for learning and memory throughout CNS development [35]. L-serine is a precursor substance of D-serine [36]. Recent studies have shown that inactivation of L-serine synthesis in astrocytes lead to a decrease in NMDAR activity, while exogenous increase in L-serine could improve cognitive ability in mice [21]. Bo et al. [37] observed that the escape latency duration of rats treated with D-serine intervention after lead exposure was markedly reduced compared to the group exposed to lead alone, with a noticeable increase in the frequency of crossing the original platform, and a concomitant rise in NMDAR-related activity, suggesting an improvement in spatial cognitive capabilities. Results of this study indicated that D-serine and L-serine concentrations in the hippocampus of offspring mice treated with arsenic significantly reduced. However, D-serine levels increased markedly after CBIO intervention, suggesting that arsenic may inhibit D-serine and L-serine concentrations while CBIO could affect D-serine levels. Results reported by Coyle and Balu [38] indicated that D-serine synthesis is mainly completed under the catalysis of SR, which specifically converts L-serine to D-serine, and the content of D-serine is also affected by SR levels. At the same time, SR has a bidirectional catalytic effect, while L-serine can be converted to D-serine, D-serine can also be converted back to L-serine. Since SR has a high selectivity for L-serine, the main direction of specific catalysis by SR is from L-serine to D-serine. Therefore, in this study, administration of DAAO inhibitor CBIO increased D-serine levels, but did not affect L-serine levels through the reverse catalytic effect of SR. Data of this study also demonstrated that levels of SR mRNA and protein in arsenic exposure group significantly reduced, suggesting a decrease in SR levels might reduce the synthesis of D-serine, and thus participate in regulating the damage to learning and memory of mice caused by arsenic. Otherwise, no obvious alterations were found in SR mRNA and protein levels of mice treated with arsenic and CBIO, indicating that CBIO intervention could not affect the transcriptional and translational levels of SR while affecting D-serine.

DAAO is the main enzyme for degrading D-serine. Liu et al. [39] found that in the simulated ischemia-reperfusion model group of mice, Nissl staining showed apoptosis of hippocampal neurons, cognitive impairment, and a marked increase in hippocampal DAAO expression. Results of this data showed that levels of hippocampal DAAO mRNA and protein of mice in arsenic group markedly increased, suggesting that arsenic exposure could give rise to elevated DAAO levels. Study showed that oral administration of DAAO intervention agent sodium benzoate to mice significantly increased D-serine levels and improved cognitive capabilities [40]. Nagy et al. [41] demonstrated that compound 30, a DAAO inhibitor, could increase the activity and excitability of hippocampal neurons and improve cognitive abilities. CBIO is an efficient inhibitor of DAAO. Liraz-Zaltsman et al. [27] found that a single injection of CBIO into the lateral ventricle of mice for 24 h after closed head injury notably improved their cognitive and motor abilities, reduced lesion volume, and weakened inflammatory response. At the same time, they observed a notable increase in hippocampal volume and the number of neurons in the hippocampal region of mice, suggesting that CBIO intervention has a protective effect on neurons. Our results confirmed that DAAO mRNA and protein levels obviously reduced in CBIO intervention group after arsenic exposure, which suggested that CBIO intervention could act on D-serine by inhibiting DAAO, thereby might alleviate arsenic-induced cognitive impairment. While CBIO is recognized as a potent DAAO inhibitor, its thiol-reactive moiety raises potential concerns regarding off-target effects, we will pay attention to this issue in the future.

Glu is the main excitatory neurotransmitter in CNS, and NMDAR is a ligand-gated ion channel for it. Glu is prevalent throughout all stages of brain development and is closely connected with synaptic plasticity [42]. The activation of NMDAR demands co-activation of Glu and D-serine. After D-serine combines with the glycine binding site on NMDAR, Glu can then open the ion channel coupled with NMDAR, thereby exerting its biological effects [43]. D-serine and glutamate binding is sufficient for NMDAR channel opening, which are still voltage sensitive, and blocked by Mg^2+^. Moreover, results reported by Barragan et al. [44] support a model in which D-serine availability serves to modulate NMDAR signaling and synaptic plasticity even when the NMDAR is blocked by Mg^2+^. Studies showed that during the development of the nervous system, NR1 is a functional subunit of NMDAR and exerts a major role in the efficiency and formation of synaptic transmission, and NR2 is a regulatory subunit of NMDAR that determines the functional characteristics of the receptor channel [45–47]. The NR2A and NR2B subunits are most closely related to learning and memory, interacting with calcium/calmodulin dependent protein kinase II to regulate synaptic plasticity, thereby regulating learning and memory function [48–50]. Results of this research declared that NR1 mRNA and protein levels significantly reduced in the arsenite group, and NR2A protein levels downregulated significantly. However, the levels of NR2A mRNA as well as NR2B mRNA and protein did not show significant changes, suggesting that arsenic exposure could affect the transcriptional and translational levels of NR1, with a more significant impact on the functional subunits of NMDAR. In addition, CBIO intervention prominently upregulated the levels of NR1 mRNA and protein of mice exposed to arsenite, suggesting that CBIO intervention could generate a marked impact on the transcriptional and translational levels of NR1, and then might affect NMDAR function. Elevated DAAO enzymatic activity has been demonstrated to catabolize D-serine, resulting in impaired NMDAR-mediated neurotransmission [51]. Direct application of purified/recombinant DAAO to cortical or hippocampal slices suppressed NMDAR-dependent LTP, an effect that was fully reversible upon D-serine co-application [52]. Mothet et al. [53] demonstrated that purified DAAO application attenuated NMDAR activation in both cultured neurons and acute brain slices, with concomitant D-serine administration rescuing receptor functionality. The therapeutic potential of DAAO inhibition in mitigating developmental neurotoxicity warrants further investigation, particularly for its translational applications in human cognitive disorders.

## Conclusion

Taken together, inhibition of DAAO levels could significantly increase D-serine and NR1 levels, and improve learning and memory levels, which suggested that DAAO might be involved in cognitive impairment induced by early-life arsenic exposure in mice via altering D-serine metabolism.

## Supporting information

S1 Table The primer sequence for PCR. (DOC)

S2 Table Changes in body, brain and hippocampal weights after arsenic exposure. (DOC)

S1 Fig Graphical abstract. (DOCX)

S1 File The original uncropped and unadjusted blot images. (RAR)

S2 File The data of body, brain and hippocampal weights. (DOCX)
