# Supplementary material for: The role of DAAO in cognitive impairment of offspring mice induced by arsenic exposure during early developmental stage
Source: PLoS One. 2025 Sep 29;20(9):e0333414. doi: 10.1371/journal.pone.0333414 (PMC12478938; doi:10.1371/journal.pone.0333414)
Supplement: S1 Table — (DOC) [file pone.0333414.s001.doc]

S1 Table. The primer sequence for PCR

| Gene | Primer sequences (5′-3′) | Length (bp) |
| --- | --- | --- |
| *SR* | Sense: GTAGGAGGAGGAGGAATGGTT  Antisense: TTCGGTGACAGTGAAGACATC | 243 |
| *DAAO* | Sense: TTTTCTCCCGACACCTGGC  Antisense: TGAACGGGGTGAATCGATCT | 159 |
| *NR1* | Sense: CACAGAAGTGCGATCTGGTGAC  Antisense: GGCATTGCTGCGGGAGT | 191 |
| *NR2A* | Sense: CTCTGATAATCCTTTCCTCCAC  Antisense: GACCGAAGATAGCTGTCATTTACT | 123 |
| *NR2B* | Sense: TCCATCAGCAGAGGTATCTACAG  Antisense: CCGTTGACTCCAGACAGGTT | 161 |
| *PSD95* | Sense: ATCCTGTCGGTCAATGGTGTT  Antisense: AGTCCTTGGTCTTGTCGTAGTC | 259 |
| *SYP* | Sense: TGCCAACAAGACGGAGAGT  Antisense: CGAGGAGGAGTAGTCACCAA | 142 |
| *GAPDH* | Sense: CAATGTGTCCGTCGTGGATCT  Antisense: GTCCTCAGTGTAGCCCAAGATG | 124 |
