# Supplementary material for: The role of DAAO in cognitive impairment of offspring mice induced by arsenic exposure during early developmental stage
Source: PLoS One. 2025 Sep 29;20(9):e0333414. doi: 10.1371/journal.pone.0333414 (PMC12478938; doi:10.1371/journal.pone.0333414)
Supplement: S2 Table — (DOC) [file pone.0333414.s002.doc]

S2 Table. Changes in body, brain and hippocampal weights after arsenic exposure（mean *±* SD, g, n = 6）

| Group | body weights | brain weights | hippocampal weights |
| --- | --- | --- | --- |
| control | 26.76 ± 2.30 | 0.33 ± 0.01 | 0.030 ± 0.003 |
| CBIO | 25.02 ± 3.54 | 0.32 ± 0.02 | 0.028 ± 0.004 |
| NaAsO_2_ | 24.72 ± 1.88 | 0.33 ± 0.03 | 0.029 ± 0.005 |
| NaAsO_2_+CBIO | 24.93 ± 1.40 | 0.31 ± 0.02 | 0.028 ± 0.003 |
