# Supplementary figures and images for: The role of DAAO in cognitive impairment of offspring mice induced by arsenic exposure during early developmental stage
Source: PLoS One. 2025 Sep 29;20(9):e0333414. doi: 10.1371/journal.pone.0333414 (PMC12478938; doi:10.1371/journal.pone.0333414)

**S1 Figure. Graphical abstract.**


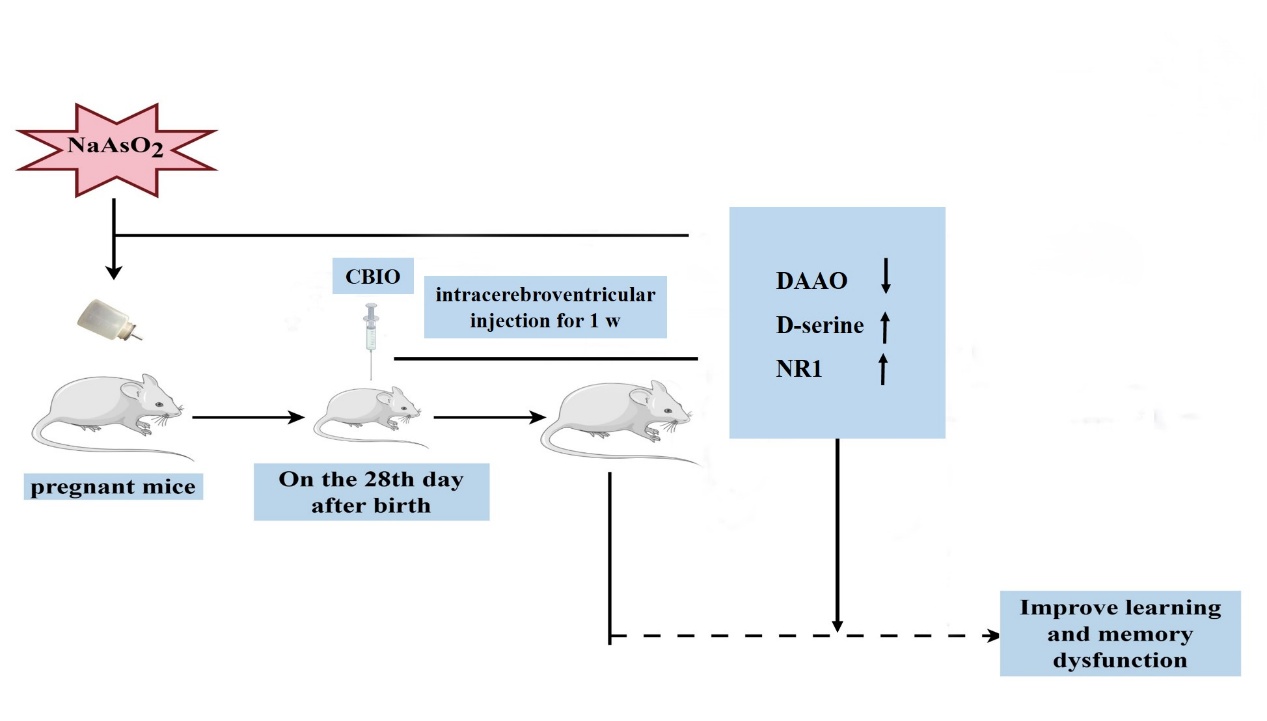

Supplement: S1 Fig — (DOCX) [file pone.0333414.s003.docx]
