# Supplementary material for: The role of DAAO in cognitive impairment of offspring mice induced by arsenic exposure during early developmental stage
Source: PLoS One. 2025 Sep 29;20(9):e0333414. doi: 10.1371/journal.pone.0333414 (PMC12478938; doi:10.1371/journal.pone.0333414)
Supplement: S2 File — (DOCX) [file pone.0333414.s005.docx]

**S2 File. The data of body, brain and hippocampal weights.**

**body weights**

| control | 26.9 | 26.8 | 28.9 | 22.5 | 26.8 | 28.7 |
| --- | --- | --- | --- | --- | --- | --- |
| CBIO | 20.7 | 21.2 | 29.2 | 28.4 | 25.0 | 25.6 |
| NaAsO_2_ | 21.1 | 26.1 | 26.2 | 24.8 | 24.7 | 25.4 |
| NaAsO_2_+CBIO | 26.3 | 25.3 | 24.1 | 24.8 | 26.4 | 22.7 |

**brain weights**

| control | 0.324 | 0.317 | 0.343 | 0.325 | 0.325 | 0.315 |
| --- | --- | --- | --- | --- | --- | --- |
| CBIO | 0.313 | 0.307 | 0.336 | 0.333 | 0.293 | 0.331 |
| NaAsO_2_ | 0.343 | 0.292 | 0.288 | 0.352 | 0.351 | 0.337 |
| NaAsO_2_+CBIO | 0.319 | 0.310 | 0.278 | 0.306 | 0.290 | 0.339 |

**hippocampal weights**

| control | 0.033 | 0.034 | 0.030 | 0.030 | 0.031 | 0.025 |
| --- | --- | --- | --- | --- | --- | --- |
| CBIO | 0.028 | 0.028 | 0.021 | 0.028 | 0.029 | 0.033 |
| NaAsO_2_ | 0.030 | 0.029 | 0.024 | 0.034 | 0.036 | 0.023 |
| NaAsO_2_+CBIO | 0.030 | 0.023 | 0.028 | 0.030 | 0.028 | 0.028 |
